# Supplementary material for: Are carbon emissions trading and green financial instruments synergistic? -Comprehensive quantitative research based on content analysis
Source: PLoS One. 2024 Mar 7;19(3):e0298601. doi: 10.1371/journal.pone.0298601 (PMC10919601; doi:10.1371/journal.pone.0298601)
Supplement: S1 Data — (ZIP) [file pone.0298601.s001.zip › Data/地级市Z_F工作报告.zip!/║╙▒▒/╒┼╝╥┐┌╩╨/└┤╫╘╣1⁄2╓┌║┼í╛┬φ┐╦╩2╛▌═°í┐_2014.docx]

各位代表：

现在，我代表张家口市人民政府向大会作工作报告，请予审议，并请市政协委员和列席会议的同志提出意见。

一、2013年主要工作回顾

2013年，新一届政府在省委、省政府和市委的正确领导下，认真贯彻党的十八大、十八届三中全会和省委八届五次全会精神，围绕“科学发展、绿色崛起”总目标，努力克服不利因素，大力调整产业结构，着力转变发展方式，全市经济社会保持了持续向好的态势。

综合实力稳步提升。全市生产总值预计完成1340亿元，同比增长8%左右；固定资产投资完成1271.9亿元，增长9.4%；全部财政收入完成224.76亿元，增长4.95%，其中公共预算收入118.47亿元，增长11.17%；全社会消费品零售总额达到494.7亿元，增长13.7%左右；城镇居民人均可支配收入预计达到20470元，增长11%左右，农民人均纯收入预计达到6290元，增长13%左右。

申奥工作有序展开。党中央、国务院批准京张联合申办2022年第24届冬季奥林匹克运动会。我市各项前期工作正式启动，组建了工作机构，完成了首轮申奥答卷，雪场建设、基础设施及产业发展等规划正在加紧编制，宣传策划、市场运作等工作全面展开。

发展后劲有所增强。全年实施亿元以上项目312个，完成投资641.8亿元；50亿元以上项目达到6个，列入省重点项目数居全省第三；100个市级重点项目完成年计划投资的106.8%，生产性项目投资比重达到51.2%。全市23个产业园区完成基础设施投资23.5亿元，新开工项目72个，总入园项目达到849个；引进了中瑞中小企业国际合作园、国家应急产业园、怀来新兴产业园。创新项目建设模式，首次参加跨国公司圆桌会议，与上海、深圳、珠海等地诸多大公司进行一对一的洽谈。与上海久有股权投资基金管理公司合作成立了张家口通泰久有产业基金，引进了泽生科技等9个高新技术产业项目。军民合用机场运行良好，京张高铁运营公司正式挂牌；国家风光储输二期工程顺利建设；沃尔沃发动机项目下线生产，凯悦汽车大部件制造开工建设。

工业强市纵深推进。全市规模以上工业企业新增130家，其中新投产企业29家，总数达到550家，完成工业增加值417.8亿元。投入技改专项资金6000万元，重点实施技改项目470个，带动企业技改投入264亿元，中煤机、盛华等一批老企业通过搬迁实现了技改扩模、产业升级。全市风电装机总容量达到600万千瓦，并网546万千瓦，继续领跑全国风电行业；以奶业、葡萄、蔬菜产业等为主的食品加工业快速发展；华美光电子、北大青鸟等一批高新技术企业正在兴起，高新技术产业增加值同比增长25.7%。

城乡面貌显著变化。中心城区完成了6756户天然气置换，北水源水处理升级改造、张电热源集中供热等工程顺利竣工，建国路地下隐患得到排除；新增绿地面积119.4万平方米，绿化覆盖率达到42.1%；新区基础设施完成投资11亿元，中央景观大道具备通车条件。县城和小城镇基础设施建设完成投资228亿元。张北、怀来两县通过国家园林县城终审，崇礼、万全两县通过省级园林县城专家组验收。实施了农村面貌改造提升五大工程，全市651个示范村的环境整治、村庄绿化基本完成，全市所有行政村实现了村村通公路。我市与大连市一起被“世界经济论坛”评为2013—2014年度新领军城市。

三农工作不断加强。粮食总产量达到166万吨，单产达到235.9公斤/亩，均创历史新高。实施重点农业产业化项目198个，市级以上龙头企业发展到309家，产业化经营率提高到62.5%。出台了扶持奶业、葡萄、蔬菜加快发展的意见，新增奶牛2.8万头，鲜奶总产量达到170万吨；新增葡萄2万亩，“宣化城市传统葡萄园”被联合国粮农组织正式批准为全省唯一的“全球重要农业文化遗产保护试点”；蔬菜种植面积和产量分别达到153万亩和671万吨。全市认定无公害农产品生产基地132万亩，认定无公害畜产品产地达到103家，55家企业、119个产品、18.6万亩基地通过绿色食品认证。流转土地190万亩，流转率达到18.3%；100亩以上的种植大户、家庭农场和农业公司发展到1093家。扶贫攻坚扎实深入开展，整合各类扶贫资金78.7亿元，建成各类蔬菜棚室16.5万个，舍饲养殖小区700多个，679个扶贫示范村基本实现致富产业全覆盖，35万人口稳定脱贫。

对外开放有所进步。全年共引进市外资金754.6亿元，增长27.2%，实际利用外资2.77亿美元，增长10.9%。与北京的合作更加深入，签约项目162项，引进资金301.9亿元，增长21.7%。全面落实《京张共建战略性新兴产业体系合作协议》，稳固了与32家央企、143家京企的合作关系，与清华大学、门头沟区、延庆县签署了全面合作协议，国电通云计算、物联网等项目落户我市。在瑞典举办了机械制造招商推介会，并与舍夫德市结为友好城市。开通了旅游包机和京张旅游专列。全年接待游客2757.4万人次，实现旅游收入185亿元，分别增长30.2%和44.5%。

生态建设继续向好。完成造林绿化135.5万亩；新增各类路网绿化925公里。“塞林精神”成为全省生态建设的一面旗帜。新增高效节水灌溉面积18.7万亩，并新建张家口灌溉试验站，全面推广张北高效节水新机制。累计关闭污染企业300多家。实施了清洁能源改造、煤炭市场关闭搬迁、建筑市场监管、渣土车运输规范管理、露天烧烤取缔等环境整治工程。全年空气质量综合指数为3.35，空气质量达标天数266天，达标比例72.9%，空气质量处于环保部监测的长江以北城市最好水平。

社会民生持续发展。深入开展“百事惠民活动”，全市用于民生领域的财政支出占到了总支出的77.13%，年初确定的126件惠民实事已基本完成。新增城镇就业5.12万人；支出各项民政事业资金20多亿元，惠及救助、救灾、优抚等民政服务对象163.7万人次；全市26.1万名企业离退人员的养老金人均月增186元；为8025名失业人员支付医保补助金909万元；新开工保障性住房1.4万套，竣工2万套，分配入住1.66万套。改造农村薄弱学校10.4万平方米；改扩建农村标准化幼儿园、增设农村小学附属幼儿园256所；列入规划的万全等6个县区全部通过省县域义务教育均衡发展评估；市教育局被国务院授予“全国两基工作先进单位”称号；市职教中心校长汪秀丽同志被教育部授予“全国教书育人楷模”称号。实施了6个市直医院、5个县级医院、29个乡镇卫生院等一批新改扩建项目；新农合参合率达到96.5%，13家县级公立医院改革试点全部实行药品零差率销售。全市乡镇文化站实现全覆盖，在全省率先实施了“一县一品”文化品牌创建活动；蔚县暖泉镇被中国旅游协会评为“2013美丽中国十佳旅游镇”；张北元中都、阳原泥河湾获国家考古遗址公园立项；三祖题材电视剧《英雄时代》在涿鹿完成拍摄，器乐合奏《古道乡音》荣获第十届中国艺术节“群星奖”；成功组织了“情系燕赵——两岸文化联谊行”大型文化交流活动。深入开展了打黑除恶、食品药品安全隐患大排查、安全生产打非治违等专项行动，有效遏制了重特大安全事故、恶性治安案件和食品药品安全事故的发生；市县党政领导干部接访包案、化解疑难信访案件，集中解决了一大批群众反映的实际问题；处置公共事件的能力和水平有所提升，和谐稳定的发展局面更加巩固。

政风行风得到改善。认真落实中央八项规定，市本级处置违规公务车108辆，清理整改办公用房使用面积2.2万平方米，“三公”经费较上年压减了15%。市本级取消和下放行政审批事项24项，减少审批环节180个，取消和免征经营性服务性收费、行政事业性收费15项。自觉接受市人大的法律监督、工作监督和市政协的民主监督，广泛听取各民主党派、工商联和无党派人士意见建议，办理人大代表建议84件，政协提案334件，按时办复率达到100%。

在肯定成绩的同时，我们也清醒地认识到，2013年全市生产总值、固定资产投资、全部财政收入、社会消费品零售总额4项指标没有达到预期目标。我市经济总量小、产业结构不合理、市场主体少、县域经济薄弱、政府债务沉重等问题没有得到根本改变。我们必须正视这些问题，本着对人民群众高度负责的态度，加倍努力地去解决这些问题。

二、2014年总体要求和工作安排

今年，京津冀协同发展和京张联合申办冬奥会，成为我市两个重大的机遇。本顺书记要求我们深入领会四个词、十六个字：“绿色崛起、调整结构、改革创新、普惠民生”，这为我们做好政府工作提出了更高、更明确的要求。我们要紧紧围绕市委确定的“转变发展理念，推进改革创新，坚持真抓实干，加速绿色崛起”工作主题，坚持“稳中求进”主基调，围绕“进”字做好文章，积极进取，奋力突破，在新的历史起点上创造更加辉煌的业绩!

2014年政府工作的指导思想是：全面贯彻落实党的十八大、十八届三中全会、中央经济工作会议、中央城镇化工作会议、中央农村工作会议、省委八届六次全会和市委十届五次全会精神，以推动经济提质增效升级为目标，以改革创新为主线，以项目建设为支撑，以发展奥运、园区、生态、县域“四型经济”为抓手，更加注重推进经济结构调整，更加注重统筹协同发展，更加注重保障改善民生，更加注重解放思想、转变作风，加快张家口绿色崛起进程。

2014年主要预期目标是：全市生产总值增长8.5%；固定资产投资增长18%；公共财政预算收入增长10%；全社会消费品零售总额增长13%；城镇居民人均可支配收入和农民人均纯收入分别增长10%和12%；实际利用外资和进出口均增长8%；单位GDP能耗下降3%，化学需氧量、氨氮、二氧化硫、氮氧化物排放量分别下降1.2 %、2.7%、1.2%和5.6%，PM2.5下降4%；城镇登记失业率控制在4.6%以内。

2014年，重点做好以下十项工作：

(一)全力以赴做好申奥工作。京张联合申办2022年第24届冬季奥林匹克运动会，是我市经济社会发展中的头等大事。紧紧围绕“2014年7月入围候选城市、2015年7月赢得主办权”这一阶段性目标，力争一次申办，一次成功。抓紧完善各项规划。以雪场为核心，编制《张家口市承办2022年冬奥会总体发展规划》，统筹做好重大基础设施建设、生态环境建设、农村面貌改造提升等专项规划，切实完善雪场整体规划设计。重新编制《崇礼县城乡总体规划》和综合交通、森林绿地、公共服务等八个专项规划。加快实施重点工程。率先启动雪场互通道路、云州水库调水主管网和主蓄水池等工程。加快雪场的新改扩建工作。加快推进京张高铁崇礼延伸线的前期工作。启动怀来到崇礼的交通主干线的通道绿化和雪场绿化工程。大力营造申奥氛围。强化对内宣传，推动群众性冰雪运动，形成全市上下支持申奥、服务申奥、参与申奥的浓厚氛围。开展文明礼仪教育，全面提升市民素质。以申奥树形象、以申奥促开放、以申奥聚人气、以申奥增实力，把申办过程变成一个引进项目、吸引投资、加快发展的过程。

(二)全面深化对外开放战略。促进与北京同城化发展。强化与北京各级政府、科研院所、医院学校的全面对接，促进与央企京企的战略合作。重点在高新技术、新兴产业等方面，进一步增强对接深度、加大对接力度、拓展对接广度。落实京张共建战略性新兴产业体系合作协议，以东花园京北生态新区为平台，吸引更多的北京企业、项目、人才落户我市。提升精细化招商水平。以小团组形式，瞄准国内外著名公司和高科技企业，进行一对一拜访洽谈。注重研究上市公司动态，积极承接扩张性投资。招聘选派一批专业人员，分批次、带任务赴经济发达城市开展驻点专职招商。充分发挥企业的招商主体作用，依托协会商会、代理投资公司，以商招商、以会招商、有偿招商。健全完善张家口招商网，增强在线互动功能，提高招商实效性。努力拓展合作领域。组织好到瑞典各大城市的招商，面向欧美国家，开展城市形象和招商引资宣传推介。深化与沿海发达地区的城市间合作。探索组建“国际知名企业家俱乐部”，组织知名企业家到我市交流合作、考察体验。

(三)把项目园区建设作为经济工作的主旋律。把大项目作为核心之举，集中力量推进一批打基础、惠当前、利长远的大项目。市级重点抓好100个前期和100个在建项目。加快推进中国版“迪斯尼”—特维乐园、高档花卉生产观光基地及宝石、动漫、电玩设计生产基地等一批新兴产业项目的前期工作，争取年内开工建设。加快沃尔沃发动机、凯悦汽车大部件、云计算等重大项目建设进度。推进总投资36亿元的君乐宝食品科技园项目，确保一期3万吨婴幼儿配方乳粉生产线4月份开工建设。力争珠海银通储能模块基地项目早日落地。谋划推进京尚、京蔚高速等重大基础设施项目，确保京张高铁上半年开工。每个园区至少引进1—2个龙头项目。做大做强南山汽车产业园、西山机械制造园、张北经济开发区、怀来新兴产业示范 区等重点园区，加快建设中瑞中小企业国际合作园、国家应急产业园。在新区谋划“硅谷”模式的高科技新城项目。推广“企业领建、市场办园”模式，引进战略投资者，以独立开发、合作经营等方式建设和经营园区，提升园区开发水平。年内市财政筹措专项资金，支持项目建设和园区公司化运作。

(四)坚定不移推进工业转型。把推进工业结构调整，加快转型升级作为主攻方向，推动工业经济向科技含量高、经济效益好、资源消耗低、支撑能力强迈进。加快技改升级步伐。大力实施“1+1”工程，每家规模以上工业企业年均实施一项技改工程，力争50个项目列入省千项技改计划。主要采取财政贷款贴息和补贴的办法，重点支持100项对调结构、转方式影响重大的技改项目。筛选50—80家有成长潜力的重点企业，与国内外大企业进行“点对点”合作。力争更多企业进入省“三个一百”工程，促进企业整体上水平。年内确保新增规模以上工业企业100家以上。大力发展新兴产业。重点推动科技研发、先进制造、新型能源、农产品精深加工、文化旅游、商贸物流、金融服务和养老医疗八大产业加快发展。积极争取滑雪服装和器具生产落户我市。探索“基金+项目”、“科技+项目”、“人才+项目”的新模式，打造经济发展的“引爆点”。市财政筹措专项资金，投向高新技术产业，以撬动产业基金、投行、实体企业等社会资本进入。增强创新驱动能力。加大科技成果引进力度，集中打造影响力大、转化水平高的科技创新孵化器。发挥好市内各类高等院校、科研院所服务经济发展的作用。继续深化与国家、省及北京市研发中心、科研院所的密切合作，吸引重大科技成果在我市转化。重点培育和扶持100家科技型中小企业。

(五)持之以恒改善生态环境。以打造国内空气质量一流城市为目标，在绿色城市、绿色产业、绿色乡村、绿色环境、绿色生活上争当全省的排头兵。强力推进大气污染防治。实施最严格的节能降耗减排措施，重点做好减煤、控车、降尘等工作。坚决取缔高速公路及国省干道两侧的煤炭市场，6月底前全部搬迁到专业园区进行封闭生产经营。今年主城区新增集中供热290万平方米，拆除和改造燃煤锅炉192台；完成省下达的钢铁、煤炭、水泥的削减任务，大力开展四大行业污染治理行动，年内全部完成治理工作；报废黄标车17706辆，主城区公交车全部更换为新能源车型。谋划推进新能源示范城市建设，以储能模块、相变储热材料为重点，利用5—10年时间力争实现清洁能源全覆盖，年内各选择一个城市小区、企业、村庄先行试点。着力构筑首都生态屏障。发扬“塞林精神”，实施好国家京津风沙源治理二期、坝上120万亩防护林更新改造工程。加强与北京在生态建设领域的合作。做好景区、通道、村庄绿化工作，推进国家森林城市创建步伐。全年完成各类林业生态建设工程130万亩，通道绿化1000多公里，建设100个绿化示范村。全面实施生态修复工程。市域境内的露天矿山，实行洞采或关闭；取缔高速公路和国省干道两侧的采石场、小砖窑。完成水土流失治理面积240平方公里。新增高效节水灌溉面积20万亩，坝上地区节水灌溉面积达到100万亩，保持坝上地下水位持续回升。

(六)积极稳妥推进新型城镇化。以“人的城镇化”和“城的产业化”为核心，以申办奥运为动力，打造与国际接轨的、具有张家口特色的新型城镇化。坚持主城区、县城、小城镇、农村“四位一体”，实行经济社会发展、城乡、土地利用规划“三规合一”，构筑层级合理、多点支撑、良性互动的城镇发展格局。对新区的发展方向进行重新定位，加快打造集高新科技、教育培训、总部经济、商贸流通、运动休闲为主体功能的生态新城。进一步完善中心城区公共服务及配套设施建设。按照“精细化、智能化、人性化”的理念，推进智慧城市建设。把县城建设成为拉动县域经济、转移农村人口的龙头。全面放开县城落户限制，力争通过三至五年的努力，把一半以上的县城都建成10—20万人口规模的小城市。加快培育发展特色产业体系，实现产业向园区集聚、园区向城镇布局，推进工业化和城镇化的融合发展。

(七)加快发展现代农业。坚持脱贫致富与全面小康同步走，大力提高农村综合发展水平，实现农业生产增效、农村活力增强、农民收入增加。强力扶贫攻坚。重点推进产业扶贫。创新扶贫模式，推广宣化县常峪口村“支部+协会+农户”、阳原县小关村“股份合作”等扶贫成功经验，探索符合本地实际、行之有效的扶贫模式。围绕“一村一品、多村一业”的连片开发格局，投入各类资金60亿元，重点打造40条扶贫主导产业带，带动贫困群众增收致富，年内确保20万贫困人口稳定脱贫。扩大对外合作。把农业资源作为一个重要的基础资源进行对外招商合作。在推进中荷现代农业综合示范项目建设的基础上，积极争取与其他北欧农业强国的合作，谋划引进农业新品种和先进农业生产机械设备。加强与国内先进地区交流合作，引入特色优势项目，增强全市产业化经营实力。推动除“原创”作物杂交谷子外的燕麦、黍子等优势农产品走向世界。增强科技支撑。着眼提升农业综合竞争力，把农业先进实用技术应用到籽种更新，耕作方式、经营方式转变等各个环节。重点扶持蔬菜、奶业、葡萄、马铃薯等特色主导产业上档升级。支持市农科院筹建市级蔬菜种苗中心，鼓励雪川集团自建科研中心。以建设奥运食品基地为目标，确保农副产品“绿色、安全、优质”，年内认定无公害蔬菜基地10万亩、无公害畜产品产地20个，认证有机农产品5000亩。推进规模经营。进一步加快土地流转，年内新增土地流转面积25万亩，新增100亩以上规模种植大户或农业公司200个。扶持农民专业合作社、龙头企业、家庭农场、专业大户等发展规模经营和精深加工，打造覆盖面广、带动力强、效益明显的产业链条。市级以上龙头企业达到350家，产业化经营率达到63%。发展农业园区。通过支农资金整合和涉农项目“融合”，每个县要谋划建设1—2个具有规模和示范效应的现代农业示范园区，年内创建10个千亩以上现代农业示范园区。重点建设以马铃薯综合加工、蔬菜加工流通为主的农业综合示范园区，以君乐宝为主的食品科技园区，以地方特色农业品种为重点的特色种养园区等三类园区建设。同时，以环首都县、京张道路沿线、雪场周边村庄为重点，进一步改善农村面貌，今年巩固提高71个重点村，改造提升350个省级重点村。

(八)不断提升服务业水平。加快发展旅游服务业。以“建设中国冰雪旅游城市”为目标，着力打造冰雪旅游特色品牌，建成以崇礼为核心、辐射带动华北地区2亿多人的滑雪区域。重点打造旅游产业的“黄金三带”，即以坝头草原天路为主体的“最美风景带”，以泥河湾、三祖、古民俗等为主题的“历史文化旅游带”，以滑雪、温泉、草原、葡萄(酒)品游为重点的“休闲体验带”，并通过科学合理规划，与北京的精品旅游线路有机地衔接，使京张旅游融为一体。加快发展现代物流业。实施大商贸、大物流、大市场战略，通过兼并收购、联合重组，对现有的企业自建物流、个体私营物流进行有效整合，组建物流商会。引进国内外大型物流企业，特别是吸引北京的物流项目入驻我市。培育引进一批电子商务龙头，着力构建现代电子商务流通体系。引导和支持内外贸企业向现代营销模式转变，引领区域消费方式转型升级。探索发展连锁式商业，加快培育连锁配送中心。大力扶持集装箱运输，降低物流成本。年内开展综合保税区申报工作。加快发展金融服务业。做好民生银行、中信银行等域外金融机构的引进工作，引导现有各家银行加快网点建设、扩大投放规模，支持村镇银行、小额贷款公司、贷款担保机构加快发展，逐步实现四大国有商业银行和市商业银行县域全覆盖、村镇银行和小额贷款公司县域全覆盖。吸引更多的专业基金公司在我市设立产业基金。谋划推进金融广场项目，以引进北京金融机构为重点，形成金融机构的集聚。推动有条件的企业到资本市场融资，力争年内企业上市实现历史性突破。

(九)大力推进全民创业。放开市场准入。建立负面清单制度，吸引民间资本投资基础设施、基础产业、社会服务等领域。实行投资主体“零限制”，注册登记“零收费”，由“先证后照”改为“先照后证”，废除影响全民创业的一切不合理规定，为市场主体大开绿灯。扶持高校毕业生、科技人员、失业人员等各类人员创业，鼓励企业兴办创业家园，扶持科研院所建立实验室和孵化器，吸引外企建设归国留学人才创业园，带动更多人员创业就业，年内力争新增1万个以上的民营经济单位。搭建创业平台。加快建设中小企业公共服务平台和创业辅导基地，为创业者提供“通用孵化楼”和标准厂房，并给予最大限度的优惠。建立全民创业综合协调服务机构，对创业者免费提供创业指导、项目评估等全方位服务。落实资金保障。加大对全民创业的资金扶持力度。大力发展融资担保机构，市县财政按照5—10%的比例注入资本金。对创业者进行每笔担保金额不超过50万元的融资支持，并根据吸纳就业情况给予一定的贷款贴息。评选创新能力强、带动就业多、社会影响好的创业带头人，市财政给予一次性奖励。支持骨干企业拓展经营领域，对投资新兴产业的给予重点扶持。加大政府采购政策支持，提高采购市内民营企业产品及服务的比例。

(十)更加注重改善民生。全面发展教育事业。大力实施“中心城区义务教育学校标准化提升工程”，三年内新建小学7所，初中2所，搬迁、改扩建高中1所、初中3所、小学4所，使中心城区教育布局明显优化，解决入学难、上学远、班容量大等突出问题；实施农村薄弱学校改造等八大类教育工程，提高县域教学水平；探索集团化、联盟化、一体化办学模式，破解义务教育均衡发展难题；加快普通高中多样化、特色化发展；打造以主城区学校为龙头的职教集团；支持张家口学院进一步发展，加快新建和搬迁步伐；继续实施“蛋奶工程”和国家营养改善计划；谋划推进国际学校建设。推动更高质量就业。逐步构建城乡均等的就业服务体系，打造张家口高校毕业生创业孵化基地，确保高校毕业生总体就业率保持在85%以上。着力抓好困难职工、零就业家庭和下岗失业人员等各类群体的就业工作，城镇零就业家庭动态为零，年内确保完成各类培训2.8万人次，转移农村劳动力就业3万名。加快社保体系建设。实施基本医疗保险市级统筹，企业退休人员基本养老金提高10%以上；新农合人均筹资标准达到390元，政策范围内住院费用支付比例达到75%以上。继续推行儿童白血病等20类重大疾病保障试点工作。大力推进城市居家养老服务中心和农村互助幸福院建设。保障困难群众生活。主城区、县区和农村低保年保障标准分别提高到5040元、4680元和2250元。农村五保集中和分散供养标准分别提高到5000元和2600元。完善社会救助和保障标准与物价上涨挂钩联动机制。做好惠民蔬菜直销、春节肉菜供应等工作。年内解决31万农村人口饮水不安全问题。加大住房保障力度。新建保障性住房14000套，竣工10000套，分配入住9100套，改造农村危房2万套。做好特殊群体供热供气、住房租赁等补贴发放工作。提升医疗卫生服务。巩固提升13家县级公立医院综合改革试点。实施90个卫生项目，进一步加强医疗卫生体系建设。实施基本公共卫生、重大公共卫生项目，推进公共卫生服务均等化。全面落实计划生育政策。大力推进文化惠民。实施市图书馆、博物馆、档案馆新建工程，加快县区文化场馆、乡镇文化站和基层综合性文化服务中心建设，推动四级公共文化服务网络的标准化和全覆盖。推进农村电影放映、文化场馆免费开放等文化惠民活动。实施市政便民工程。继续加大“三网”建设力度，加快供热计量改造步伐，主城区住宅供热计量改造面积完成700万平方米。完成盛华东大街供水管网、主城区老旧燃气管网等改造工程。加快发展公交事业，新开通公交线路2条，优化线路1条。确保食品药品安全。全面落实食品药品安全监管制度，用最严谨的标准、最严格的监管、最严厉的处罚、最严肃的问责，确保全市人民群众“舌尖上的安全”。促进社会和谐稳定。持续开展好安全生产、消防安全、交通安全、打黑除恶、大气污染等专项治理行动。加快气象自动监测站网建设，提升气象灾害防御能力。深化信访制度改革，健全和完善源头预防、会诊评议等制度。今年，要突出办好十件具体实事，通过认真筛选研究、广泛征求各方意见确定后，由政府各职能部门全力抓好落实。

三、切实加强政府自身建设

(一)简政放权，着力转变政府职能。全面清理市本级行政审批事项，按照“取消一批、下放一批、合并一批”的原则，年内市级行政审批事项压减三分之一以上。清理并逐步取消市级各部门非行政许可审批事项，消除审批管理中的“灰色地带”。公开市级全部行政审批事项清单，接受社会监督，防止边减边增、明减暗增。对保留的行政审批事项，规范行政行为，简化审批程序，优化审批流程，最大限度减少预审和前置审批环节。彻底改变“收费代替管理”的做法，严禁下达罚没任务目标，严禁罚没收入与单位经费及个人收入挂钩。做好经营性服务性收费、行政事业性收费的免征和取消工作。今年不再增加新的财政供养编制。探索增强乡镇活力的改革。做好市、县机构改革有关工作。开展政府向社会力量购买服务试点工作。

(二)廉洁从政，不断改进工作作风。深入开展第二批党的群众路线教育实践活动，严格落实中央八项规定。进一步强化行政监督和问责，着力解决工程建设、土地出让、产权交易、政府采购等方面的腐败问题。认真落实党风廉政建设责任制，严格执行领导干部廉洁从政的各项规定。继续开展社会评议政府职能部门活动，对排名末位的部门或单位由监察部门予以预警，并由市政府领导对其主要负责人进行诫勉谈话。加大明察暗访力度和频次，对存在“庸、懒、散”等现象的各类人员通报曝光，视情节给予处分。按照“零容忍”的标准，严肃惩处不作为、乱作为的“害群之马”。搞好“文山会海”治理工作，建立文件、会议、活动计划编报和审批制度；坚持少而精、短实新原则，压缩文件简报篇幅；健全完善“无会月”制度，把更多时间用于抓落实；坚决撤销、停办不符合国家有关法律法规及中央、省、市有关文件规定的节庆庆典活动；健全完善文件、会议、活动经费预决算制度，严控计划外和预算外经费使用。年内市级“三公”经费压减15%以上。

(三)依法行政，推进科学民主决策。完善重大行政决策程序，严格落实决策前须经专家论证、征求公民意见、集体讨论研究等规定，并对决策实施实行全方位监督，最大限度地规避风险和失误。全面落实行政执法责任制，切实做到有法必依、执法必严、违法必究。自觉接受市人大及其常委会的法律监督、工作监督和市政协的民主监督，支持监察、审计部门依法独立监督。广泛听取社会各界的意见和建议，重视发挥新闻媒体和社会公众监督的作用。

各位代表!面对新的形势、新的任务，我们深感责任重大，使命光荣。让我们在市委的坚强领导下，抢抓机遇，创新突破，狠抓落实，推动经济社会发展再上新台阶!
